# Case report: Gross total resection of a primary fourth ventricular meningioma using the telovelar approach in a dog

**DOI:** 10.3389/fvets.2024.1450332

**Published:** 2024-08-22

**Authors:** Jaemin Jeong, Haebeom Lee, Yoonho Rho, YoungJin Jeon

**Affiliations:** ^1^Department of Veterinary Surgery, College of Veterinary Medicine, Chungnam National University, Daejeon, Republic of Korea; ^2^Department of Veterinary Surgery, College of Veterinary Medicine, Gyeongsang National University, Jinju, Republic of Korea

**Keywords:** meningioma, fourth ventricle, fourth ventricular meningioma, telovelar approach, dog

## Abstract

An 11-year-old spayed female Maltese dog presented with a 2-month history of gait alterations, wide-based stance, and chronic vomiting. Neurological examination revealed cerebellovestibular signs, including head tilt, nystagmus, strabismus, intentional tremor, and hypermetric gait. MRI showed a mass with iso- to hypointensity on T1-weighted (T1W) images and heterogeneous hyperintensity on T2-weighted (T2W) images, with marked non-uniform contrast enhancement. The tumor was removed via a telovelar approach without intraoperative complications. Postoperatively, the dog developed non-ambulatory paraparesis with the rigidity of the pelvic limbs but recovered ambulation within 6 days. Preoperative neurological signs progressively improved, and the patient was discharged without complications 10 days after surgery. Histological examination revealed dense spindle cells with an abundant collagen matrix and oval-shaped nucleated cells with small whorls, leading to a diagnosis of transitional meningioma of the fourth ventricle. MRI follow-up at 8 months postoperatively showed no definitive evidence of recurrence. At the final follow-up, 15.4 months postoperatively, mild neurological signs, including a slight head tilt and subtle strabismus, remained, but the rest of the neurological examination was normal. This is the first reported case of a meningioma in the fourth ventricle of a dog successfully removed using the telovelar approach.

## 1 Introduction

Primary ventricular tumors are relatively uncommon in veterinary medicine. The most common tumor located in the ventricular system is the choroid plexus tumor (CPT), which accounts for up to 7% of primary brain tumors and is the third most common type of primary intracranial tumor in dogs (1, 2). Other primary tumors, such as ependymoma, astrocytoma, and meningioma, can also occur within the ventricles, although reported cases are limited (3–9). The prevalence of primary fourth ventricular tumors is presumed to be less than 5% of all primary brain tumors. This estimation is based on the finding that 49% of CPT are located in the fourth ventricle, while other primary fourth ventricular tumors have been reported only rarely as single-case reports (7, 10). The clinical signs associated with fourth ventricle tumors are predominantly cerebellovestibular signs, including strabismus, head tilt, obtunded mental status, and delayed postural reactions (7, 11). Additionally, an absent menace response has also been reported (11).

Meningiomas typically occur in the rostrotentorial region, accounting for 62.2 to 84.4% of all cases (12, 13). In contrast, intraventricular meningiomas are extremely rare; only two lateral ventricular meningiomas and one-fourth ventricular meningioma have been described (6, 7, 9). The single case of a fourth ventricular meningioma was diagnosed postmortem through histopathologic examination and classified as a microcystic subtype, which is a rare form of meningioma. In human medicine, intraventricular meningioma is similarly rare, constituting 0.7–3% of intracranial meningiomas, with only 6.6% of these occurring in the fourth ventricle (14, 15).

While a standard treatment protocol for fourth ventricular tumors has not been established in veterinary medicine, surgical removal is considered the first line of treatment in human medicine (16). The surgical approach to the fourth ventricle is particularly challenging due to its narrow anatomical location between the brain stem, specifically the medulla oblongata and the cerebellum (17). In the human literature, the transvermian and telovelar approach are well-described methods for accessing the fourth ventricle. The telovelar approach has been used to surgically resect a choroid plexus carcinoma (CPC) within the fourth ventricle in a dog (11). This technique offers several advantages over the transvermian approach, such as sparing the vermis and providing better exposure in craniocaudal and lateral directions (11, 18, 19).

The author found no existing literature describing the surgical removal of a fourth ventricular meningioma and its outcome in veterinary medicine. This case report aims to describe the neuroradiological findings, detailed surgical technique, and long-term outcome of a primary fourth ventricular meningioma, classified as the transitional subtype.

## 2 Case description

**Figure 1:**
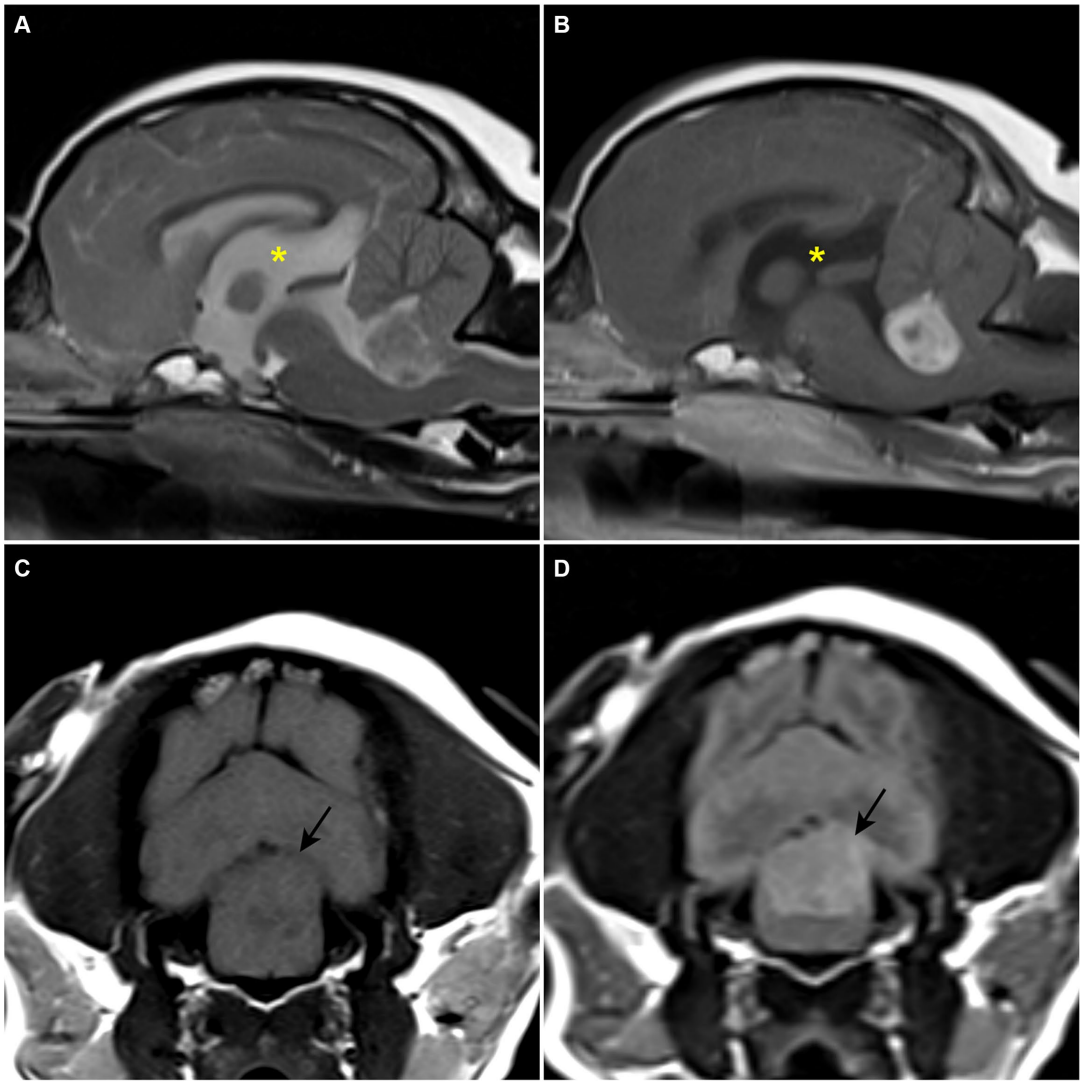
Preoperative MRI. **(A)** T2-weighted sagittal plane. A heterogenous hyperintense space-occupying the lesion is confirmed within the fourth ventricle. **(B)** T1-weighted post-contrast sagittal plane. A strong enhanced mass-like lesion is confirmed within the fourth ventricle. An enlarged third ventricle (asterisk) is revealed. **(C)** T1-weighted transverse plane. Hypo- to isointense lesion is confirmed. **(D)** T2 fluid-attenuated inversion recovery transverse plane. The lesion is revealed as a hyperintensive signal.

A client-owned, 11-year-old spayed female Maltese dog weighing 3.8 kg was referred with a 2-month history of falling episodes with gait alterations, wide-based stance, nausea, and vomiting. Nausea and vomiting could not be controlled by maropitant (1 mg/kg, orally, q24hr) at the primary veterinary clinic. The physical examination revealed a systolic murmur of grade 4/6 at the left heart apex, which was later evaluated as grade B1 myxomatous mitral valve disease; other findings were normal. Neurological examination revealed bright and alert mentation, an obvious left head tilt, positional, horizontal nystagmus with fast phase to the right, ventrolateral positional strabismus of left eye, intentional head tremor, and hypermetric gait. Concurrent vestibular and cerebellar signs indicated a lesion on the left central vestibular system and cerebellum. The lesion was thought to be a space-occupying mass, and differential diagnoses included neoplasia (CPT, glioma, meningioma, and lymphoma), cyst, or cyst-like lesion. Inflammatory diseases, anomalies, and degenerative diseases were thought to be less likely. Blood tests were within normal reference range, except for a mildly elevated liver panel. No abnormal findings were identified on the thoracic radiographs. MRI revealed a 10.4 × 11 × 16.2 mm sized, oval-shaped, and slightly left-sided intraventricular mass. The mass showed iso- to hypointensity on T1W images, heterogeneous hyperintensity on T2W images, hyperintensity on fluid-attenuated inversion recovery sequences (FLAIR), and marked non-uniform contrast enhancement (Figures 1A–D). The mass compressed and displaced the medulla oblongata ventrally and the cerebellum dorsally. Obstructive ventriculomegaly was observed rostral to the lesion. The neuroradiological findings corresponded with the symptoms. A presumptive diagnosis of an intraventricular tumor was made, with CPT considered the most likely differential diagnosis.

Surgical removal of the tumor using the telovelar approach was planned as described in a previous study (11). Prior to the anesthetic induction, maropitant (1 mg/kg, IV), dexamethasone (0.2 mg/kg, IV), and cefazoline (22 mg/kg, IV) were administered. The patient was premedicated with midazolam (0.2 mg/kg, IV) and administered propofol (4 mg/kg, IV) slowly, followed by intubation with a reinforced endotracheal tube to avoid kinking due to surgical position. The dog was maintained with 1–1.5% (vaporizer setting) isoflurane with a constant 100% O2 flow of 2 L/min. The patient was positioned with the neck flexed as much as possible, slightly over 90 degrees, to achieve the maximum opening of the foramen magnum, taking care not to compress the jugular vein. After the patient preparation, mannitol (0.25 g/kg, IV) was infused slowly over 15 min to achieve better brain relaxation and prevent reperfusion injury (20–22). During anesthesia, the monitoring parameters, including electrocardiogram, heart rate (HR), respiratory rate (RR), oxygen blood saturation (SpO2), rectal temperature, non-invasive blood pressure (NIBP), end-tidal CO2, tidal volume, airway pressure, and compliance, were within the normal range. Remifentanil was administered IV at a flow rate of 5–6ug/kg/h for analgesia.

**Figure 2:**
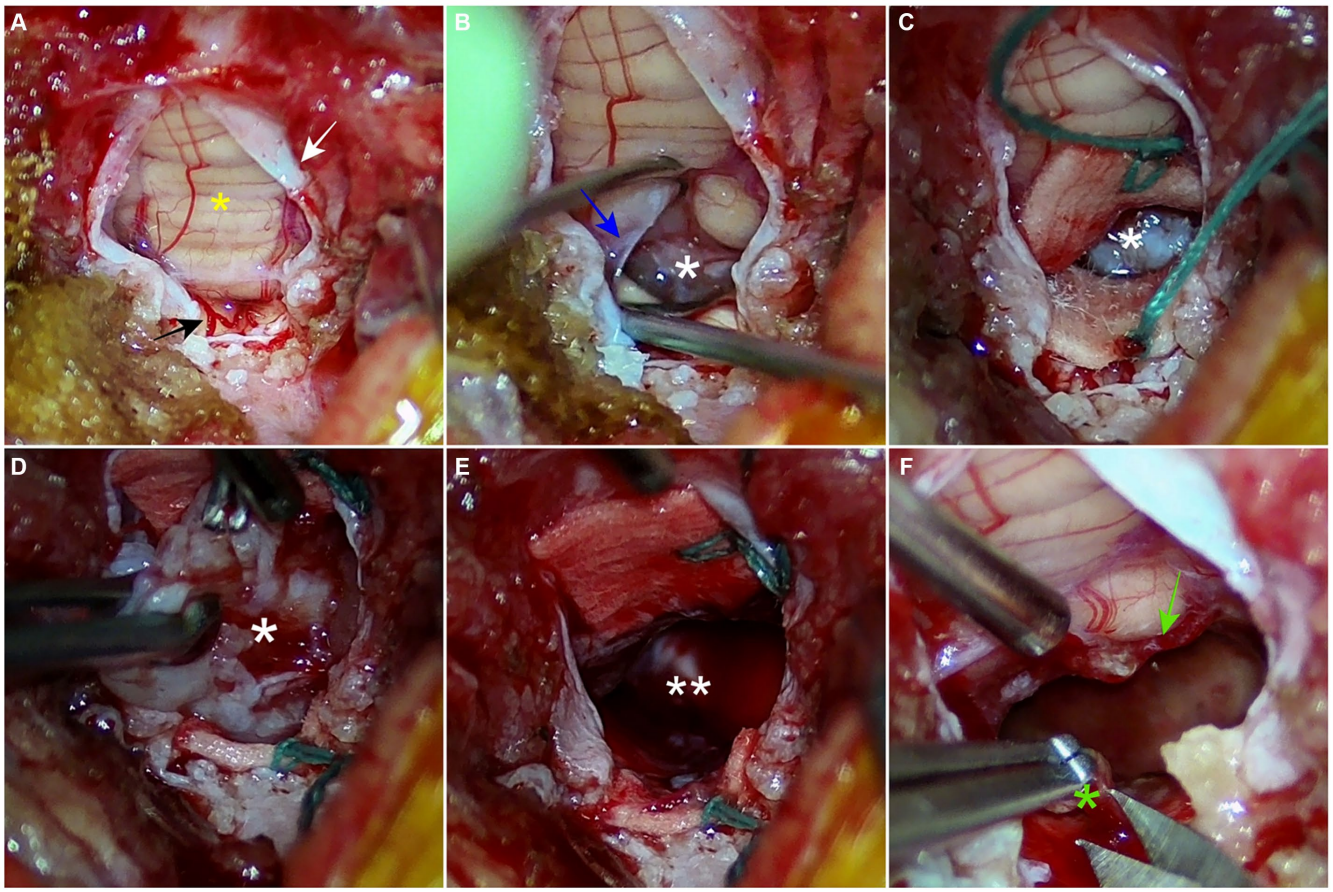
Intraoperative images from neurosurgical microscope. **(A)** A dural incision is made longitudinally from the level of the cerebellar uvula to the level of the atlas. The vermis and dura mater are indicated as a yellow asterisk and a white arrow, respectively. A branch of the caudal cerebral artery is highlighted with a black arrow. **(B)** An avascular tela choroidea (blue arrow) is incised with a nerve hook probe. The tumor (white asterisk) is confirmed through the incision. **(C)** Two green-stringed neurosurgical sponges are packed around the tumor to retract the brain parenchyma and secure the surgical field. **(D)** Through repeated meticulous maneuvers using tumor forceps, a hook probe, and a micro-ring curette, the tumor is bluntly dissected from the fourth ventricle **(E)** The tumor is removed, and the space (double white asterisks) of the fourth ventricle is confirmed. **(F)** The remaining suspected tumor capsule (green asterisk) is partially resected. The remaining choroid plexus is highlighted with a green arrow.

The surgical approach to suboccipital craniectomy was performed routinely (11, 23). Suboccipital craniectomy was performed and additional partial dorsal laminectomy of the atlas was performed to expose the widest opening of the caudal fossa. Both procedures were conducted meticulously using a Kerrison rongeur. When the caudal fossa was sufficiently exposed, a neurosurgical microscope was used to magnify the surgical site and capture the image (Figure 2). Micro-bleeding from the dura mater was coagulated by bipolar electrocautery to maintain a clear surgical site. The dura mater was picked with a double-pronged tissue pick instrument and incised with a von Graefe knife without excessive tension. The incision was made from the level of the cerebellar uvula to the level of the atlas, longitudinally. The dura mater was retracted laterally without suturing. The tela choroidea was identified as the thin, avascular, membranous layer located between the uvula and the medulla oblongata. The tela choroidea was easily incised with a blunt-ended, 90°-angled Sisson nerve hook, and the tumor was identified through the incision. Neurosurgical lint-free sponges were packed around the tumor to visualize the tumor and retract the brain parenchyma. Visible blood vessels on the tumor were coagulated with a bipolar electrocautery. The hook probe, a micro-ring curette, and tumor forceps were used alternately to perform blunt dissection around the tumor with minimal traction. Meticulous maneuvering of the cerebellum dorsally with a semi-blunt Sachs nerve elevator through the sponge provided a wider surgical site. During the dissection, minor bleeding was controlled with sponges and ophthalmic triangular swabs. After the dissection, the mass was detached from the ventricle by grasping it with tumor forceps and removed as a single solitary piece. Minor bleeding after removal was controlled by attaching absorbable oxidized regenerated celluloses to the ventral surface of the ventricle and filling the ventricle with Hartmann’s solution. Sponges were removed and generous irrigation was performed to confirm the absence of residual bleeding and to spare the ventricle space. A thin sheath, thought to be a residual tumor capsule, was confirmed on the caudodorsal surface of the medulla oblongata and partially removed with Castroviejo scissors. The tela choroidea remained open, and the dura mater was sutured with a synthetic dura substitute (ReDura, Medprin Biotech, Frankfurt, Germany) to maximize the space over the foramen magnum using 6/0 absorbable suture material (6/0 PDS II, Ethicon, Raritan, NJ) in a single interrupted pattern. Closure of the muscle layer and skin was performed routinely. The entire removed mass was submitted for histopathological examination.

**Figure 3:**
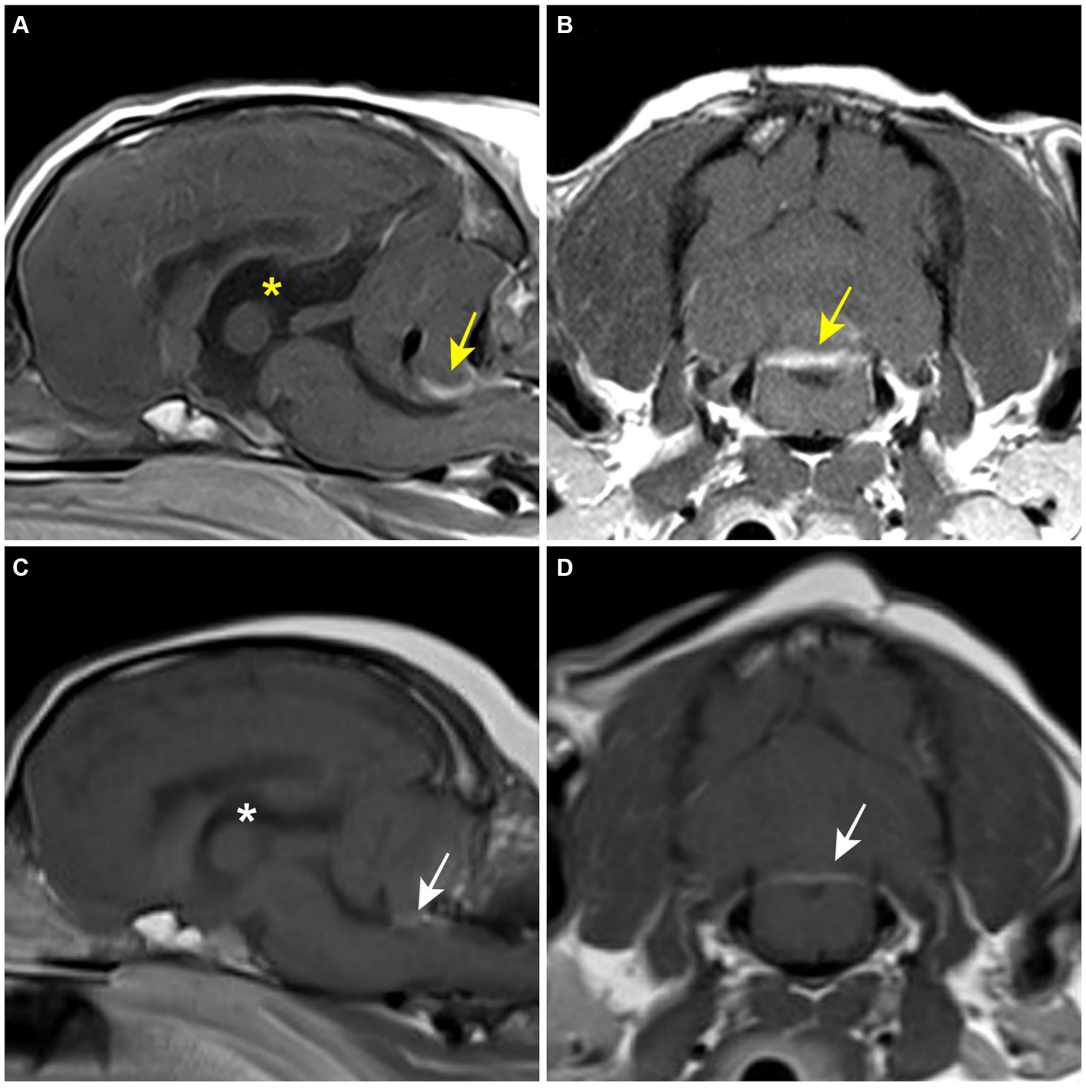
Postoperative MRI. **(A,B)** Immediate postoperative T1-weighted post-contrast MRI. A contrast-enhanced lesion (yellow arrow) is revealed on the caudoventral aspect of the cerebellum. The third ventricle (yellow asterisk) is still enlarged. **(C,D)** 8-month postoperative T1-weighted post-contrast MRI. Contrast-enhanced lesion (white arrow) and the third ventricle (white asterisk) are reduced compared to the former MRI findings.

**Figure 4:**
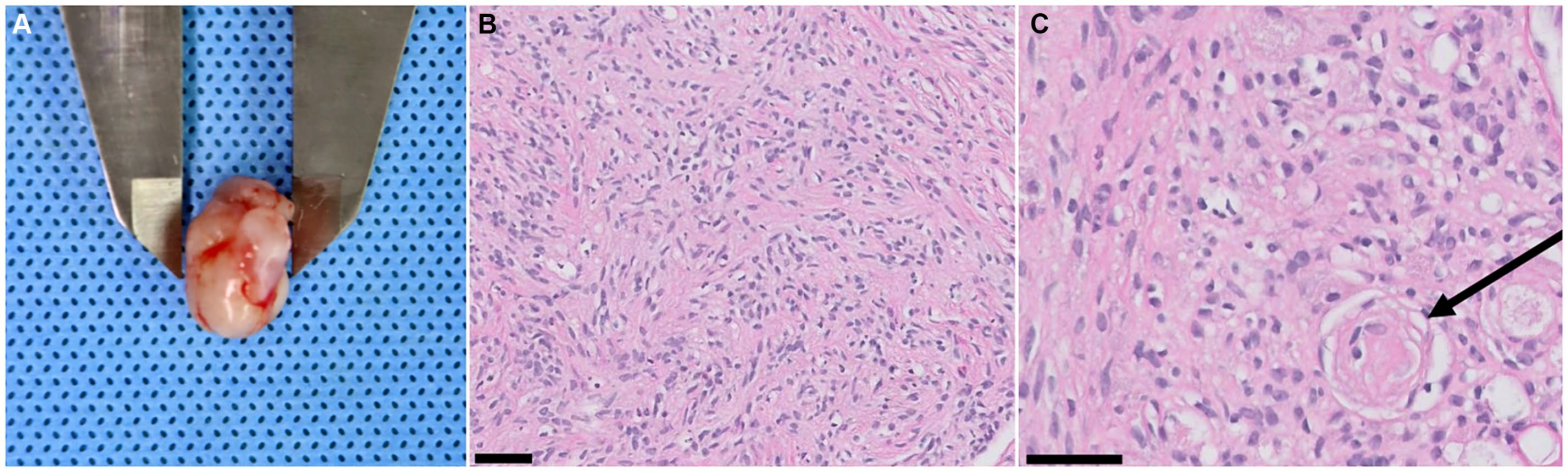
Tumor gross appearance and biopsy results. **(A)** A resected fourth ventricular tumor. The gross appearance is white to gray (relatively low vascularization), oval-shaped, 15.8 × 10.5 × 10 mm in size, and has a firm texture. **(B)** Dense spindle cells with an abundant collagen matrix are confirmed. These findings are typical findings of the fibrous subtype of meningioma (magnification ×20, black 50 μm scale bar, hematoxylin and eosin stain). **(C)** Oval-shaped nucleated cells with small whorls (black arrow) are revealed, indicating the meningothelial subtype meningioma. (magnification ×40, black 50 μm scale bar, hematoxylin and eosin stain).

The patient was moved for a postoperative MRI to confirm the surgical removal of the tumor. Immediate postoperative MRI revealed the removal of the tumor, but a strongly enhancing structure was confirmed ventral to the caudal cerebellar lobe on T1W with gadolinium images (Figures 3A,B). The tumor was white to gray, oval-shaped, 15.8 × 10.5 × 10 mm, and firm (Figure 4A). Histological examination revealed dense spindle cells with an abundant collagen matrix, the typical finding of the fibrous subtype, and oval-shaped nucleated cells with small whorls, the common finding of the meningothelial subtype (Figures 4B,C). The combination of the findings, including medical imaging, gross appearance, and histopathologic findings, led to a definitive diagnosis of primary fourth ventricular transitional meningioma.

The patient recovered smoothly from anesthesia. Vital signs were monitored and neurological examinations were performed at regular intervals throughout the convalescent period. There was no significant alteration of vital parameters, such as HR, PR, NIBP, and body temperature, during monitoring. On the day of the surgery, non-ambulatory paraparesis with hindlimb rigidity was observed but improved after a single infusion of mannitol (0.5 g/kg, IV, slowly over 15 min). A single event of mental dullness was observed 3 days after the surgery, but it soon recovered after a single administration of mannitol (0.5 g/kg, IV, slowly over 15 min). Preoperative signs, such as vomiting and vestibular signs, improved progressively, and ambulation recovered 6 days after the surgery. During hospitalization, amoxicillin-clavulanic acid (20 mg/kg, IV, q12hr), prednisolone (0.5 mg/kg, PO, q12hr), levetiracetam (20 mg/kg, IV, q8hr), gabapentin (10 mg/kg, PO, q12hr), esomeprazole (1 mg/kg, IV, q12hr), and maropitant (1 mg/kg, IV, q24hr) were administered. Acetazolamide (10 mg/kg, PO, q8hr) was added after the event of mental dullness. Remifentanil was infused (6ug/kg/h, IV) for analgesia, gradually tapered according to the monitored pain response, and withdrawn at 3 days post-surgery. The patient was discharged 10 days after surgery with remaining neurological signs, including positional nystagmus, slight head tilt, and hypermetria. The antibiotic was discontinued and hydroxyurea (50 mg/kg, orally, every other day) was added to the medication upon discharge.

At the first follow-up, 43 days after surgery, the patient showed head tilt, positional nystagmus, and hypermetria, but other clinical signs had resolved. Medication was modified to exclude acetazolamide and reduce the dosage of prednisolone to 0.3 mg/kg, q12hr. At 6-month postoperative follow-up, neurologic signs recover to normal, except for a slight head tilt. Levetiracetam was discontinued, and prednisolone was reduced to 0.5 mg/kg, q24hr. Eight months after the surgery, a follow-up MRI was requested by the client to verify no recurrence of the tumor. The former contrast-enhanced structure had reduced significantly and appeared to be a membranous structure (Figures 3C,D). The size of the lateral ventricle returned to within the normal reference; preoperative ventricle/brain index: 0.61, 8-month postoperative ventricle/brain- index: 0.56 (24). There was no recurrence of the mass-like lesion in the fourth ventricle. At the final follow-up at 15.4 months postoperatively, the patient showed a slight head tilt and subtle positional strabismus. The client was satisfied with the outcome. Throughout the follow-up, regular blood work, including complete blood count, total protein, globulin, alanine aminotransferase (ALT), alkaline phosphatase (ALP), aspartate aminotransferase and gamma-glutamyl transferase, was performed every 2 months to monitor the effect of the medication, especially prednisolone and hydroxyurea. Mild anemia and elevated ALT and ALP were observed throughout the medication but without further deterioration. Based on the MRI findings and the absence of further deterioration in neurological signs, it was presumed that there was no recurrence of the tumor.

## 3 Discussion

This case report describes the first successful surgical removal of a primary fourth ventricular transitional meningioma in a dog, providing a detailed account of MRI findings, surgical technique, postoperative care, and outcome. Despite the uncommon occurrence of intraventricular meningiomas in dogs, it is essential to consider meningioma in the differential diagnosis for fourth ventricular masses due to their differing characteristics from CPTs.

The recurrence of the brain tumor is closely related to the completeness of the resection. Although there are limited cases of surgical treatment of fourth ventricle tumors, meningiomas are thought to be more feasible to resect totally in a gross manner than CPT. In our case, the tumor was well-encapsulated and relatively less vascularized. Therefore, it was able to be removed en bloc without piecemeal maneuvering and with minimal bleeding. A previous study described that a CPT was removed in three pieces and the figures showed that the sponge was notably red, indicating more bleeding from the tumor resection compared to our case (11). Another report, describing the removal of lateral ventricular CPT in a dog, reported bleeding as the main intraoperative complication (25). Additionally, human literature indicates that uncontrollable hemorrhage during CPT resection is associated with perioperative mortality, which can range up to 30% (26). Although it is difficult to draw definitive conclusions from a single case, these characteristics can impact surgical outcomes. Therefore, further research through a larger number of case studies on a fourth ventricular meningioma is necessary to better understand the prognosis related to surgery.

Preoperatively, CPT was the primary consideration due to its neuroanatomical prevalence and histological origin. The choroid plexus consists of pia, ependyma, and proliferated capillaries and is found within the ventricular system (17). CPTs, including choroid plexus papilloma (CPP) and carcinoma (CPC), originate from the choroid plexus epithelium (10, 27). In contrast, meningiomas typically arise from arachnoid cap cells (1). To date, only four cases of intraventricular meningiomas have been reported in the veterinary literature, including one case of primary fourth ventricular microcystic meningioma, two cases of lateral ventricular meningioma, and the current case (6, 7, 9). Although studies on the origin of intraventricular meningiomas in veterinary medicine are limited, human literature suggests they may arise from the stroma of the choroid plexus or tela choroidea (28). The diagnosis of meningioma in our case adds further evidence that meningiomas can indeed occur in this location within the ventricular system of dogs.

MRI is considered the gold standard for diagnosing brain diseases. However, differentiating between CPC and intraventricular meningioma using MRI is challenging. Some pathological differences may be reflected in MRI findings (29). CPCs are highly vascular, with abundant microvasculature, making them more likely to appear hypointense on T1W, hyperintense on T2W, and exhibit signal voids on FLAIR (30). Additionally, their gross appearance can be indicated by an irregular margin on MRI images (31). According to previous veterinary studies, these MRI features of CPT align with the pathological hypotheses (1, 10, 13, 32–34). In our case, the tumor showed iso- to hypointensity on T1W, heterogeneous hyperintensity on T2W, hyperintensity on FLAIR, and strong enhancement after gadolinium infusion. However, the few reported intraventricular meningioma cases, including our case, showed significant variation in MRI findings (6, 7, 9). Therefore, MRI alone is not reliable for differentiating between CPTs and intraventricular meningiomas. Further studies are needed to correlate MRI findings with histological characteristics to improve diagnostic accuracy.

In our case, postoperative MRI sequences confirmed that the tumor was removed and showed no signs of recurrence at 8 months postoperatively. An initial contrast-enhanced lesion observed on the immediate postoperative MRI was found to have reduced in size on the 8-month postoperative MRI. Differential diagnoses for this lesion included residual choroid plexus, inflammation, and remaining tumor tissue. The choroid plexus was considered the most likely due to the surgical findings, the lesion’s size reduction, and prolonged use of prednisolone. The choroid plexus, located beneath the caudal lobe of the cerebellum and extending into the lumen of the fourth ventricle, can enhance with contrast due to its rich blood supply and lack of a blood–brain barrier (17, 35). Furthermore, a residual sheath, presumed to be the tumor capsule, was identified on the caudodorsal surface of the brainstem, which did not correspond to the lesion’s location. However, there remains a possibility of residual neoplastic cells in the choroid plexus since intraventricular meningiomas can originate from it. Thus, regular follow-ups to monitor neurological status and two postoperative MRI scans have been implemented.

The temporary deterioration of neurological signs observed post-surgery, including non-ambulatory paraparesis and mental dullness, may be attributed to retraction and maneuvering during the procedure, as well as to reperfusion damage and subsequent edema following decompression Additionally, it could be a result of acute postoperative hydrocephalus. According to human literature, residual hemostatic material is known to be a risk factor for acute postoperative hydrocephalus since it can cause occlusion of cerebrospinal fluid flow (36, 37). The interaction between cerebrospinal fluid, blood, and foreign bodies tends to promote coagulation (38). Predictive factors for acute hydrocephalus following lateral ventricular tumor resection include preoperative hydrocephalus and the presence of intraventricular hemostatic agents, both of which were relevant in our case (39). Therefore, meticulous handling and protection of the parenchyma during surgery, along with generous irrigation with warm fluid, are crucial for preventing complications after the fourth ventricular tumor resection (36). Additionally, careful monitoring and immediate treatment are essential for managing postoperative complications (40).

This case report has several limitations. First, it is based on a single case, thus further studies are needed to verify MRI findings for diagnosing intraventricular tumors and to evaluate the prognosis of surgically resected intraventricular meningiomas. Additionally, the pathomechanism of mental dullness observed 3 days after the surgery could not be explained, as MRI or CT images were not taken at the time. Finally, although there was no evidence of recurrence according to neurological examination and MRI at the final follow-up, the risk of recurrence remains. Therefore, further investigation of the long-term outcomes is necessary.

In conclusion, this case report presents the first instance of a surgically treated primary fourth ventricular meningioma in a dog, providing a detailed account of MRI findings, surgical technique, postoperative care, and its satisfactory outcome. This case highlights the importance of including meningioma in the differential diagnoses for fourth ventricular masses, despite the rarity of intraventricular meningiomas in dogs.

## Data Availability

The raw data supporting the conclusions of this article will be made available by the authors, without undue reservation.
